# Analyses of the oligopeptide transporter gene family in poplar and grape

**DOI:** 10.1186/1471-2164-12-465

**Published:** 2011-09-26

**Authors:** Jun Cao, Jinling Huang, Yongping Yang, Xiangyang Hu

**Affiliations:** 1 Key Laboratory of Biodiversity and Biogeography, Kunming Institute of Botany, Institute of Tibet Plateau Research at Kunming, Chinese Academy of Sciences, Kunming, 650204, China; 2 Institute of Life Sciences, Jiangsu University, Zhenjiang, Jiangsu, 212013, China; 3 Department of Biology, East Carolina University, Greenville, NC, 27858, USA

## Abstract

**Background:**

Oligopeptide transporters (OPTs) are a group of membrane-localized proteins that have a broad range of substrate transport capabilities and that are thought to contribute to many biological processes. The OPT proteins belong to a small gene family in plants, which includes about 25 members in Arabidopsis and rice. However, no comprehensive study incorporating phylogeny, chromosomal location, gene structure, expression profiling, functional divergence and selective pressure analysis has been reported thus far for Populus and Vitis.

**Results:**

In the present study, a comprehensive analysis of the OPT gene family in Populus (*P. trichocarpa*) and Vitis (*V. vinifera*) was performed. A total of 20 and 18 full-length OPT genes have been identified in Populus and Vitis, respectively. Phylogenetic analyses indicate that these OPT genes consist of two classes that can be further subdivided into 11 groups. Gene structures are considerably conserved among the groups. The distribution of OPT genes was found to be non-random across chromosomes. A high proportion of the genes are preferentially clustered, indicating that tandem duplications may have contributed significantly to the expansion of the OPT gene family. Expression patterns based on our analyses of microarray data suggest that many OPT genes may be important in stress response and functional development of plants. Further analyses of functional divergence and adaptive evolution show that, while purifying selection may have been the main force driving the evolution of the OPTs, some of critical sites responsible for the functional divergence may have been under positive selection.

**Conclusions:**

Overall, the data obtained from our investigation contribute to a better understanding of the complexity of the Populus and Vitis OPT gene family and of the function and evolution of the OPT gene family in higher plants.

## Background

Substrate transport is vital for all living organisms, and many transporters play important roles in this process. More than 600 transporter families are currently documented in the Transporter Classification Database (TCDB) [1,2]. These protein families are further classed into seven subclasses (channels/pores, electrochemical potential-driver transporters, primary active transporters, group translocators, transport electron carriers, accessory factors involved in transport, and incompletely characterized transport systems). In general, they have specific localizations within the cell and are specialized to carry different compounds, including nitrate, phosphate, sucrose, amino acids, peptides, hormones or metals.

The peptide transporter family consists of electrochemical potential-driven transporters that catalyze uptake of their solutes by a cation-solute symport mechanism [3]. In plants, peptide transporters can be classified into three distinct groups based on sequence similarity and mechanism of action, namely the ATP-binding cassette family, the peptide transporter family and the oligopeptide transporter (OPT) family. The plant ATP-binding cassette proteins use the energy generated by ATP hydrolysis to drive the transport of substrates such as peptides, metal chelates or glutathione conjugates [4]. The peptide transporters have been shown to transport nitrate, and di- and tripeptides [5,6]. Members of the OPT family were first characterized in yeast [7,8], and since then they have also been found in archaea, bacteria and plants. Phylogenetic analyses of plant OPT members have revealed two distant clades: the yellow stripe-like (YSL) proteins and the OPTs. The YSL transporters are involved in metal homeostasis through the translocation of metal-chelates [9-16]. The OPT proteins likely do not have a common biological function and may be involved in four different processes: long-distance metal distribution [17], nitrogen mobilization [18-21], heavy metal sequestration [19,21-23], and glutathione transport [19,21,22,24]. These processes may play a role in plant growth and development [see [25] for review].

Structurally, OPT proteins are predicted to have about 16 transmembrane strands (TMS). Through detailed bioinformatic analyses of these transporters, Gomolplitinant and Saier [26] suggested that the 16-TMS proteins might have arisen from a 2-TMS precursor-encoding genetic element that was subject to three sequential duplication events. Since the transporters are predicted to function in peptide uptake, the expansion or fusion of the TMS might make excellent physiological sense in evolution.

The structural features or expression profiles of some OPT homologs have been partially described in Arabidopsis [18] and rice [23]. Hoverer, there is much less information about this family in woody plant species such as *Populus trichocarpa *(poplar) and *Vitis vinifera *(grape). In the present study, we performed a genome-wide identification of OPT family genes in Populus and Vitis. Detailed analyses including sequence phylogeny, gene organization, conserved motifs, expression profiling, functional divergence and adaptive evolution were performed. Our results should provide a framework for further functional investigations on these genes.

## Results and Discussion

### Identification of the OPT gene family in Populus and Vitis

To identify members of the OPT gene family in Populus and Vitis, we first searched relevant databases using the corresponding Arabidopsis and rice OPT protein sequences as queries. Additional searches were also performed based on keyword querying. The Populus and Vitis sequences returned from such searches were confirmed as encoding OPTs using the CDD (Conserved Domain Database) [27,28] and Pfam http://pfam.sanger.ac.uk/ databases. As a result of this process, we identified 20 OPT genes in Poplar (Table 1) and 18 in Vitis (Table 2). The number of OPT genes present in the Arabidopsis and rice genomes was reported to be 12 and 25, respectively [18,23]. The OPT genes in Vitis and Populus encode highly hydrophobic polypeptides (grand average hydrophobicities of 0.329 to 0.628) ranging from 372 to 760 amino acids in length, with predicted pIs ranging from 5.45 to 9.44. The polypeptides were also predicted to contain from 8-16 transmembrane helices (TMHs) (Tables 1 and 2). Further analyses using the protein subcellular localization prediction software WoFL PSORT http://wolfpsort.org enabled us to predict the probable protein localization for each of the different candidate OPTs in Vitis and Populus. It was found that all candidate OPTs identified in our study are most likely to be localized in the plasma or vacuolar membranes. PtOPT1, PtOPT4, VvOPT4 and VvOPT9 had a 100% probability of being localized to the plasma membrane. For all other OPTs, although the plasma membrane was predicted as the most likely location, it is also possible that they are localized to the membranes of organelles such as the chloroplast, nucleus or Golgi apparatus (Tables 1 and 2).

**Table 1:** Oligopeptide transporter genes identified in Populus

Gene name	Gene ID	Genomic position	Protein Length	pI	GRAVY*	No. of TMHs**	PSORT predictions***
PtOPT1	7458477	LG_VI:414929..418505(-)	744	9.14	0.382	16	P: 13
PtOPT2	7476446	LG_V:6874384..6878065(+)	747	8.45	0.43	12	P: 11, N: 1, C: 1
PtOPT3	7461839	LG_II:7990890..7994069(+)	724	9.07	0.496	12	P: 8, V: 3, E.R.: 2
PtOPT4	7469682	LG_IV:5289927..5295373(+)	752	9	0.459	16	P: 14
PtOPT5	7474080	LG_XVI:102654..100307(-)	661	9.44	0.381	14	P: 9, E.R.: 3, Ch: 2
PtOPT6	7485123	LG_I:30102965..30106100(+)	756	8.92	0.396	16	P: 12, V: 1
PtOPT7	7485120	LG_I:36933302..36936452(+)	724	9.31	0.406	14	P: 9, V: 3, E.R.: 2
PtOPT8	7472512	LG_XVI:5869..8859(+)	748	9.07	0.411	12	P: 10, E.R.: 4
PtOPT9	7496418	LG_XII:2251759..2269550(+)	723	8.93	0.391	14	P: 9, V: 2, E.R.: 2
PtOPT10	7456066	LG_III:8705285..8709320(+)	750	8.06	0.411	14	P: 8, G: 3, E.R.: 2
PtOPT11	7465336	LG_III:13357805..13361193(+)	760	5.57	0.392	14	P: 9, C: 3, M: 1
PtYSL1	7467032	LG_I:5980774..5978118(-)	662	9.13	0.596	14	P: 8, G: 3, V: 2
PtYSL2	7477824	LG_I:5980705..6181638(-)	652	9.23	0.526	14	P: 10, E.R.: 2, V: 1
PtYSL3	7464319	LG_XII:2602881..2601171(-)	625	9.29	0.615	11	P: 6, Ch: 4, E.R.: 3
PtYSL4	7492083	LG_V:2731980..2734897(+)	665	9.16	0.524	15	V: 8, P: 3, G: 2
PtYSL5	7494364	LG_IV:2622178..2624820(-)	687	8.86	0.426	12	P: 10, G: 2, V: 1
PtYSL6	7495797	LG_I:24263574..24268468(+)	669	5.54	0.628	13	P: 8, V: 4, G: 2
PtYSL7	7470849	scaffold_184:5062..8519(+)	668	9.13	0.496	12	P: 7, V: 4, G: 3
PtYSL8	7470829	scaffold_184:6206..5052(-)	372	9.09	0.565	8	G:5, P:4.5, G:4.5, Ch:2, N:1, V:1
PtYSL9	7483743	scaffold_5835:114..2350(+)	582	8.89	0.497	9	P: 8, V: 3, G: 2

*GRAVY means Grand average of hydropathicity.

** Number of transmembrane helices predicted with TMHMM Server.

*** PSORT predictions: P (plasma membrane), V (vacuolar membrane), C (cytosol), Ch (chloroplast), N (nuclear), E.R. (endoplasmic reticulum), M (mitochondrion) and G (Golgi apparatus).

**Table 2:** Oligopeptide transporter genes identified in Vitis

Gene name	Gene ID	Genomic position	Protein Length	pI	GRAVY*	No. of TMHs*	PSORT predictions*
VvOPT1	100242147	Chr18:6409616..6406367(-)	757	6.51	0.46	14	P: 12, C: 1
VvOPT2	100247286	Chr18:6415993..6411758(-)	754	8.61	0.377	16	P: 12, V: 1.
VvOPT3	100242155	ChrUn:18477250..18484183(+)	715	8.78	0.359	14	P: 10, C: 2, V: 1
VvOPT4	100250922	Chr3:10834213..10825455(-)	744	8.59	0.438	14	P: 13
VvOPT5	100267883	ChrUn:18469581..18480735(+)	533	9.25	0.371	9	P: 10, E.R.: 3
VvOPT6	100249762	Chr19:8962812..8965954(+)	745	8.7	0.435	16	P: 8, C: 3, E.R.: 2
VvOPT7	100257243	Chr16:21603624..21599997(-)	722	9.33	0.46	14	P: 12, E.R.: 1
VvOPT8	100259035	Chr18:6402500..6398183(-)	759	8.91	0.43	15	P: 7, V: 4, E.R.: 2
VvOPT9	100251260	Chr2:2104857..2109137(+)	752	5.61	0.434	14	P: 13
VvOPT10	100256645	Chr19:8994304..8997460(+)	753	8.95	0.399	14	P: 7, C: 4, E.R.: 2
VvYSL1	100242270	Chr1:1967827..1970932(+)	718	8.71	0.452	14	P: 8, E.R.: 4, V: 2
VvYSL2	100249683	Chr17:14986220..14981193(-)	708	7.44	0.355	14	P: 9, V: 2, G: 2
VvYSL3	100255514	Chr16:21498741..21495037(-)	655	8.99	0.508	14	P: 10, V: 2, E.R.: 2
VvYSL4	100256406	Chr17:14425936..14430684(+)	704	8.77	0.347	14	P: 10, G: 2, V: 1
VvYSL5	100252983	Chr17:14475366..14480298(+)	708	8.36	0.329	14	P: 9, V: 2, G: 2
VvYSL6	100260063	Chr14:25417683..25408791(-)	649	5.74	0.588	12	V: 9, P: 4
VvYSL7	100244078	Chr1:1989810..1993774(+)	713	8.95	0.347	14	P: 8, E.R.: 4, V: 2
VvYSL8	100233121	Chr2:2172478..2175411(+)	661	9.04	0.498	14	P: 10, V: 2, E.R.: 2

*Notes of GRAVY, TMH and PSORT are shown in Table 1.

### Phylogenetic analyses, classification and functional relatedness of the OPT genes in Arabidopsis, rice, Populus and Vitis

**Figure 1:**
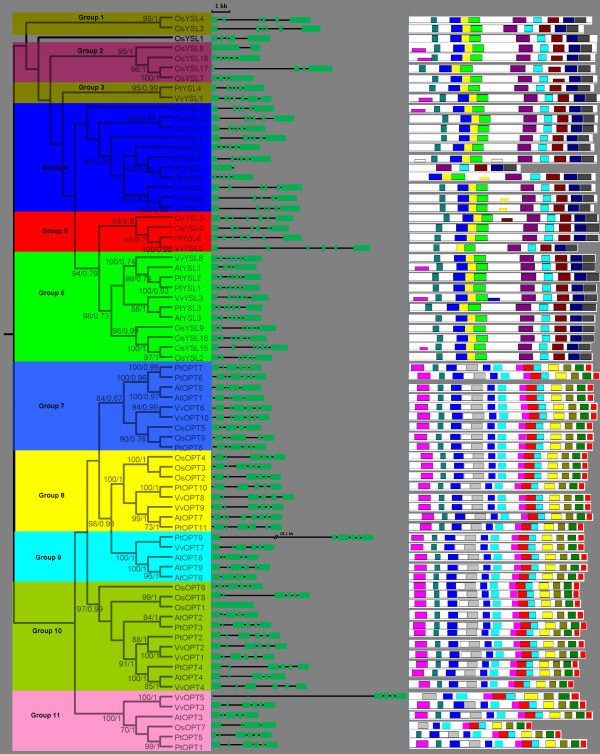
**Phylogenetic relationships, gene structure and motif composition of OPT genes in Arabidopsis (At), Populus (Pt), Vitis (Vv) and rice (Os)**. The molecular phylogeny (left panel) was constructed using full length OPT protein sequences from the four species. Numbers associated with branches show bootstrap support values for maximum likelihood analyses and posterior probabilities for Bayesian analyses, respectively. The 11 major groups designated from 1 to 11 are marked with different color backgrounds. Exon/intron structures of the OPT genes are shown in the middle panel. Green boxes represent exons and black lines represent introns. A schematic representation of conserved motifs (obtained using MEME) in OPT proteins is displayed in the panel on the right. Different motifs are represented by different colored boxes. Details of the individual motifs are in additional file 5: Sequence logo and regular expression of the different motifs identified in the OPT gene family.

To examine the phylogenetic relationships among the OPT genes in Arabidopsis, rice, Populus and Vitis, we performed phylogenetic analyses of the OPT protein sequences from all four species based on a maximum likelihood method using PhyML 3.0 [29] and Bayesian analyses using PhyloBayes 3 [30]. Our results show that the OPT genes from the four higher plants consist of two major clades: the OPT and YSL classes. In this study, we further divide the YSL class into six subclasses according to their phylogenetic relationships and they are designated as Groups 1-6. The OPT class is also further divided into five subclasses, namely Groups 7-11 (Figure 1). The relationships of OsYSL1 with other OPT genes, however, cannot be confidently determined in our analyses: OsYSL1 was basal to a large clade consisting of Groups 2-6 with weak support in the maximum likelihood analyses, but formed a clade with Group 1 in Bayesian analyses. Therefore, OsYSL1 is not classified into any group in this study. Most of the designated groups are supported by decent bootstrap values and/or posterior probabilities. Moreover, other lines of evidence, such as gene structure and motif compositions as described below, also support the group classification in our analyses. Groups 4, 6 and 10 constitute the largest clades in the OPT phylogeny, each containing 11 members. Additionally, Groups 5 and 6 form a well supported clade in the maximum likelihood analyses, suggesting that they likely evolved from a common ancestor by frequent gene duplication.

Genes with same functions often are closely related and this has been confirmed in previous reports [18,23,31,32]. Such a trend is also found in the OPT genes. For instances, Group 4 includes the AtYSL1 and AtYSL3 proteins, both of which are involved in metal ion homeostasis and the loading of metal ions in seeds [33,34]. AtYSL1 and AtYSL3 proteins also have dual roles in reproduction: their activity in leaves is required for normal fertility and normal seed development, while their activity in inflorescences is required for proper loading of metals into seeds [35]. Another member in this group, OsYSL2, has metal-nicotianamine transport activities in heterologous expression systems [12]. AtOPT6, a member of Group 9, is able to transport glutathione derivatives and metal complexes under sulfur-deprived conditions and may be involved in stress resistance, whereas AtOPT7 of Group 8 is not involved in stress resistance [19,21]. The high AtOPT6 expression reported in the vasculature of roots, stems and leaves also suggests that this protein is involved in long-distance peptide transport or distribution throughout the plant [19,20].

Phylogenetic analyses can allow us to identify evolutionarily conservative and divergent OPT genes. Remarkably, Groups 1 and 2 do not include any Arabidopsis, Vitis or Populus OPT proteins but contain only proteins from rice. Likewise, Group 9 does not include any rice OPT proteins but contains only proteins from Populus, Vitis and Arabidopsis. It is possible that these groups have evolved after monocot-dicot divergence and that they have specialized roles in monocots or dicots. Our phylogenetic analyses also show that Groups 4 and 5 contain sequences from rice, Vitis and Populus but not from Arabidopsis, indicating that they were either acquired in rice, Vitis and Populus or lost in Arabidopsis. Although enormous evidences indicates that all these OPT genes encode membrane proteins that translocate their substrates from either the extracellular environment or an organelle into the cytosol, their exact functional roles are different [9-17,19,20]. The phylogenetic analyses conducted in our study may also provide potential support for their functional differentiation. Additional evidence supporting this notion comes from the tissue-specific expression profiling available on GENEVESTIGATOR [36] and the extremely different expression pattern of OPTs in rice (see additional file 1: Microarray based expression profiles of rice OPT genes across a variety of tissue or organs). For example, OsYSL15 is specifically highly expressed in rhizomes, suggesting a specific role in root development. While OsYSL1, OsYSL3, OsYSL4, OsYSL7, OsYSL8 and OsYSL11 show higher expression levels in pollen, indicating a key role in pollen development or reproduction.

Our phylogenetic analyses also show that several pairs of OPT proteins are putative paralogs (Figure 1). These putative paralogous OPT proteins account for over 44.4%, 40%, 33.3% and 40% of the entire OPT family in Vitis, Populus, Arabidopsis and rice, respectively, with sequence identifies ranging from 61% to 98% (see additional file 2: Pairwise identities between homologous pairs of OPT genes from Vitis, Populus, Arabidopsis and rice). These paralogous OPT members are closely related within the species, and have a very similar structure as described below (middle panel in Figure 1), indicating that they evolved from relatively recent gene duplications. We also estimated the evolutionary dates of the segmental duplication events using *K_s_*as the proxy for time (Table 3). Three of the four pairs (PtOPT1/PtOPT5, PtYSL1/PtYSL2, PtYSL8/PtYSL9) in Populus have very consistent *K_s _*values (from 0.24447 to 0.30110), suggesting that the duplication events in this species occurred within the last 13.43 to 16.54 million years. This period is consistent with the time (13 Ma) when a recent large-scale genome duplication event is thought to have occurred in Populus [37]. For rice, the segmental duplication event was estimated to have occurred between 27.04 to 106.11 Ma, following the divergence of monocots and eudicots (170-235 Ma). Among them, about half of the rice OPT duplication events occurred approximately when grasses originated (55-70 Ma) [38-40]. It is interesting that four of the OPT gene duplications (PtOPT6/PtOPT7, VvOPT3/VvOPT5, VvOPT10/VvOPT6, VvYSL2/VvYSL4) were estimated to have occurred more recently (only about 1.58 to 5.97 Ma). These relatively recent duplications were not found in Arabidopsis or rice. It is likely that Arabidopsis and rice have subsequently suffered a high level of gene loss [41].

**Table 3:** Inference of duplication time in paralogous pairs

Paralogous pairs	*K_a_*	*K_s_*	Data (million years ago)
VvOPT1/VvOPT2	0.1004	0.47699	36.69
VvOPT3/VvOPT5	0.03518	0.07756	5.97
VvOPT10/VvOPT6	0.0241	0.02577	1.98
VvYSL2/VvYSL4	0.01133	0.02049	1.58
PtOPT1/PtOPT5	0.03736	0.24447	13.43
PtOPT6/PtOPT7	0.01565	0.03607	1.98
PtYSL1/PtYSL2	0.12045	0.3011	16.54
PtYSL8/PtYSL9	0.05279	0.28604	15.71
AtOPT6/AtOPT9	0.10962	0.99913	33.3
AtOPT1/AtOPT5	0.12939	0.80831	26.94
OsOPT1/OsOPT8	0.13652	0.67946	52.27
OsOPT2/OsOPT3	0.0631	0.3515	27.04
OsYSL3/OsYSL4	0.17389	0.48266	37.13
OsYSL7/OsYSL17	0.29914	0.80453	61.89
OsYSL11/OsYSL12	0.1657	1.03669	79.75

### Exon-intron evolution of the OPT family genes in Arabidopsis, rice, Populus and Vitis

To investigate the mechanisms of the structural evolution of OPT paralogs, we compared the exon-intron structure of individual OPT genes in Arabidopsis, rice, Populus and Vitis. Figure 1 provides a detailed illustration of the distribution and position of introns within each of the OPT paralogs. In general, the positions of some spliceosomal introns are conserved in orthologous genes from the four lineages. In many cases, not only is the intron position shared, but the intron phase is shared as well. Moreover, the conservation of the exon-intron organization or gene structure in paralogous genes is usually strong and sufficient to reveal evolutionary relationships of introns [42]. It is clear that duplication plays an important role in the organization of genes and that intron losses have occurred frequently after segmental duplication [43]. Our study of AtOPT6/AtOPT9 and OsOPT1/OsOPT8 duplication also suggests that this mechanism underlies the evolution of these paralogs and intron losses are associated with duplications (Figure 1). The phenomenon of intron loss following gene duplication also occurred in the evolution of many other genes including the aromatic amino acid hydroxylase (AAAH) family [44]. In general, the structural diversity of gene family members provides a mechanism for the evolution of multiple gene families, while intron loss or gain can be an important step in generating structural diversity and complexity [45,46]. In this study, we analyzed the structural diversity of OPT genes and found that intron loss/gain events occurred during the expansion and structural evolution of OPT paralogs. We found that most OPT genes in the same subgroups/clades have similar coding sequences and a very similar exon-intron structure, strongly supporting their close evolutionary relationship. The divergent gene structures in the different phylogenetic subgroups may represent gene family expansion from ancient paralogs or multiple origins of gene ancestry.

### Chromosomal location of the OPT genes and duplication events in the genome

**Figure 2:**
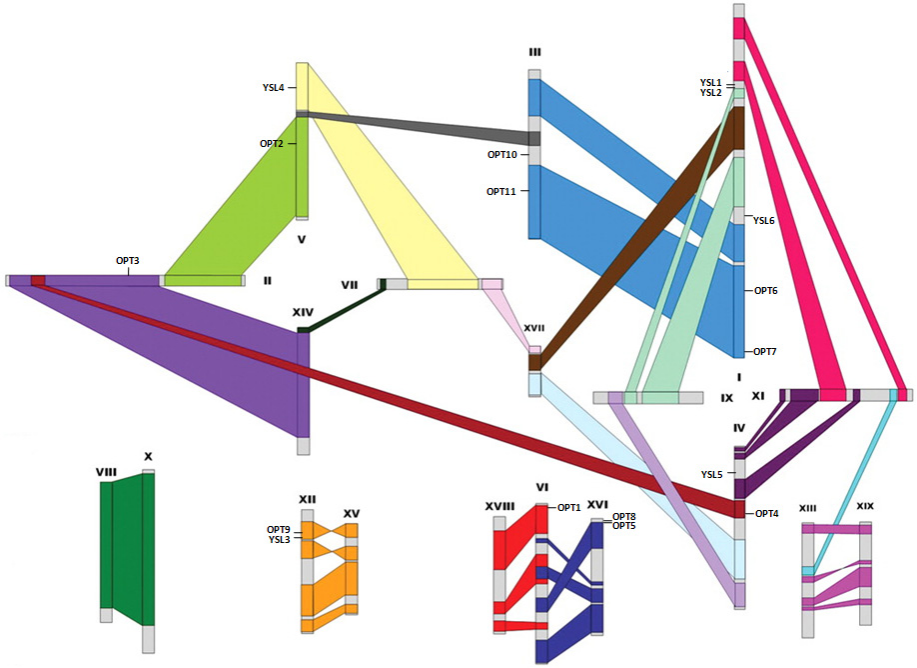
**Chromosomal locations of the Populus OPT genes**. The schematic diagram shows the 17 OPT genes mapped to 8 chromosomes. Three remaining genes (PtYSL7, PtYSL8 and PtYSL9) are located on unassembled scaffolds. Homologous blocks derived from segmental duplication are indicated using the same colors. The diagram of genome-wide chromosome organization resulting from genome duplication events in Populus is adapted from Tuskan *et al*. (2006) [49].

**Figure 3:**
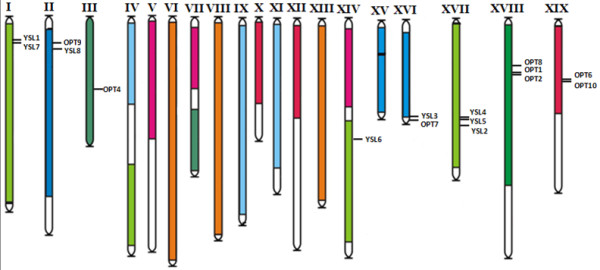
**Chromosomal locations of the Vitis OPT genes**. The 16 OPT genes mapped to the 8 of the 19 grape chromosomes are shown. Two remaining genes (VvOPT3 and VvOPT5) are located on unassembled scaffolds. Paralogous regions in the putative ancestral constituents of the Vitis genome are depicted using the colors according to Jaillon *et al*. (2007) [41] and Licausi *et al*. (2010) [50].

Genome-wide duplication events, gene loss and local rearrangements have created the present complexities of the genome. To further investigate the relationship between the genetic divergence within the OPT family and gene duplication and loss in the Populus and Vitis genomes, we determined the chromosomal location of each OPT gene. The results show that the OPT genes are dispersed throughout the Populus and Vitis genomes. Three of the Populus OPT genes are localized to unassembled genomic sequence scaffolds and thus could not be mapped to any particular chromosome. The other OPT genes are distributed unevenly among the eight chromosomes of the Populus genome (Figure 2). Five OPT genes were identified on chromosome I, two on each of chromosomes III, IV, V and XII, and only one on each of chromosomes VI and II. For Vitis, 16 OPT genes were found on 8 of the 19 chromosomes; three on each of chromosomes XVII and XVIII, two on each of chromosomes I, II, XVI and XIX, and one on each of chromosomes III and XIV (Figure 3). Two other Vitis OPT genes could not to be assigned to a specific chromosome.

Gene duplication events are thought to have frequently occurred in organismal evolution [47,48]. To investigate the relationship between the OPT genes and potential gene duplications within the genome, we also compared the locations of OPT genes in duplicated chromosomal blocks that were previously identified in Populus, Vitis, Arabidopsis and rice [41,49-52]. The distribution of the OPT genes relative to the corresponding duplicated chromosomal blocks is illustrated in Populus (Figure 2), Vitis (Figure 3), Arabidopsis (see additional file 3: Chromosomal locations of the Arabidopsis OPT genes) and rice (see additional file 4: Chromosomal locations of the rice OPT genes). This result suggests that segmental duplication and transposition events are not the major factors that led to the expansion of the OPT gene family in the four higher plants. It may be that dynamic changes occurred following segmental duplication, leading to loss of many of the genes. Interestingly, we found that some OPT genes are located in tandem clusters on the chromosomes; examples are PtYSL1-PtYSL2, PtOPT8-PtOPT5, AtOPT9-AtOPT8, OsYSL7-OSYSL8, OsYSL2-OsYSL15, OsYSL9-OsYSL16, OsYSL3-OsYSL4, OsOPT2-OsOPT3 and VvOPT1-VvOPT2-VvOPT8 (Figure 2 and 3; see also additional file 3: Chromosomal locations of the Arabidopsis OPT genes and additional file 4: Chromosomal locations of the rice OPT genes). Further analyses indicate that most of the tandemly clustered OPT pairs share relatively high similarities (mostly above 70%). Thus, we propose that tandem duplications might have been an important factor governing the expansion of the OPT gene family in these species.

### Conserved domains and motifs in OPT proteins

The major domains of the OPT proteins in Populus, Vitis, Arabidopsis and rice were identified using CDD, Pfam and SMART [27,28]. Our results show that all OPT proteins in the four species possess only one characteristic and structurally conserved OPT domain essential for their transporter activity. While these tools are suitable for defining the presence or absence of recognizable domains, they are unable to recognize smaller individual motifs and more divergent patterns. Thus, we further used the program MEME [53] to study the diversification of OPT genes in Populus, Vitis, Arabidopsis and rice. Twenty distinct motifs were identified in these genes (Figure 1). Details of the 20 motifs are presented in additional file 5: Sequence logo and regular expression of the different motifs identified in the OPT gene family. As mentioned above, phylogenetic analyses broadly divided the OPT genes from the four higher plants into two major classes, the OPT class and the YSL class. Noticeably, most of the closely related members in each of these two main classes have common motif compositions, suggesting functional similarities among the OPT proteins within the same class (Figure 1). Most members of OPT class possess 14 motifs, while most members of YSL class have 9 motifs. Three of the motifs (motif 1, motif 2 and motif 7) are shared by all OPT proteins. Whether the motifs that are specific to the OPT class (motif 3, 4, 5, 9, 10, 12, 15, 16, 17, 18 and 19) or to the YSL class (motif 6, 8, 11, 13, 14 and 20) confer unique functional roles to the OPTs remains to be further investigated. In any case, the conserved motifs in the OPT proteins from the same class may provide additional support to results of the phylogenetic analyses. On the other hand, the divergence in motif composition among different classes may indicate that they are functionally diversified.

### Differential expression profiles of the Populus and Vitis OPT genes

**Figure 4:**
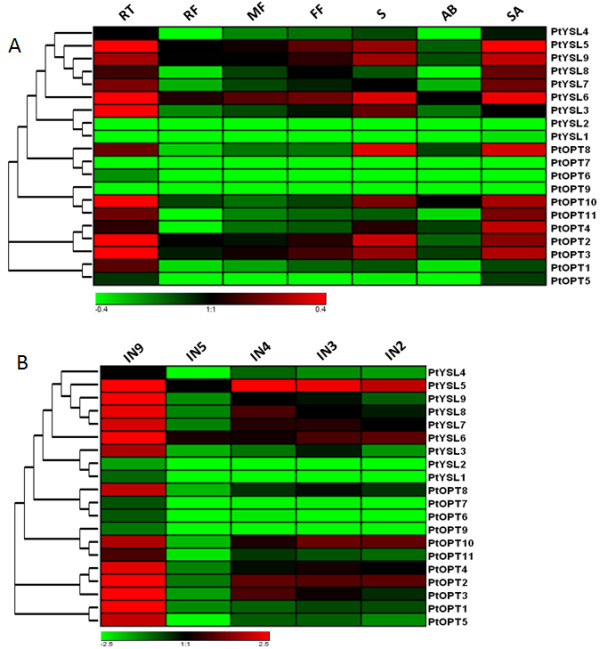
**Expression profiles of the Populus OPT genes**. **A**. Dynamic expression profiles from GEO: GSE21481 of the 20 OPT genes in different tissues. RT, roots from tissue culture; RF, roots from field trees; MF, male floral bud initials; FF, female floral bud initials; S, seedling 43 hr post-imbibition; AB, axillary buds; SA, shoot apex. **B**. Expression profiles from GEO: GSE13043 of the 20 OPTs in different internodes. IN9, internode 9; IN5, internode 5; IN4, internode 4; IN3, internode 3; IN2, internode 2.

Expression profiling can provide useful clues to gene functions. To examine the expression patterns of the OPT genes, we performed a comprehensive expression analysis using some of the publicly available microarray data for Populus and Vitis. In general, the expression levels of most OPT genes in Populus peaked in shoot apices, roots and internode 9 (Figure 4). Because these are the growing points of plants, they are likely to need more nutrients to ensure plant growth and differentiation. Because OPTs are membrane-localized proteins and have a broad range of substrate transport capabilities, higher expression of OPTs in these parts might contribute to many growth and developmental processes. Some OPTs seem not to follow this trend. For example, PtOPT10 displayed especially high expression levels in internode 2.

**Figure 5:**
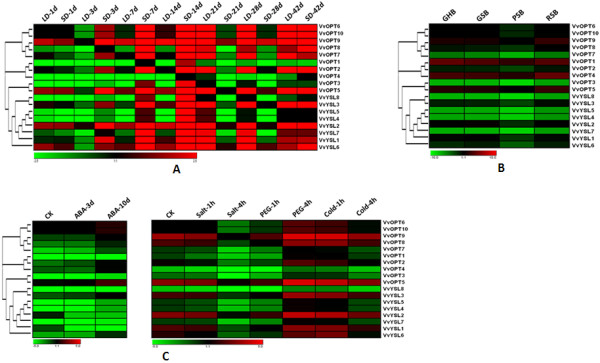
**Expression profiles of the Vitis OPT genes**. **A**. Expression patterns from GEO: GSE17502 of the 18 OPTs for different sunshine durations. LD-1d, long day (15 h) for 1 day; SD-1d, short day (13 h) for 1 day and so forth. **B**. Hierarchical clustering of the expression profiles from GEO: GSE11406 of the 18 OPT genes for different fruit development periods. GHB, green hard berry; GSB, green soft berry; PSB, pink soft berry; RSB, red soft berry. **C**. Expression profiles from PLEXdb: VV1-RMA and VV17-RMA of the 18 OPTs under different stress conditions.

Grape and wine production is strongly affected by environmental cues during the development of the plant. Here, we also investigated the expression pattern of the OPT genes in response to some abiotic stresses. Because sunshine duration can affect the quality of fruits, long daylight hours will cause grape plants to produce more carbohydrates (e.g. sucrose). Microarray data indicate that some OPTs vary considerably in their expression levels when exposed to long daylight (LD) or short daylight (SD) (Figure 5A). VvOPT2, VvYSL3 and VvYSL7 showed higher expression levels in LD compared with in SD. One possible explanation may be that, in LD conditions, grape plants need more transporters (such as the OPTs) to transport more oligonucleotide peptides for increased carbohydrate synthesis. We also examined the expression patterns of the Vitis OPTs under different stress conditions. Interestingly, several genes such as VvYSL1 and VvYSL2 showed low expression levels when treated with ABA, whereas a subset of genes including VvYSL3, VvOPT6 and VvOPT10 displayed high expression levels under salt stress (Figure 5C). Similarly, several genes such as VvOPT3 and VvYSL7 demonstrated depressed expression patterns in cold conditions. We further selected four growth phases of the fruit to investigate the different expression of the OPT genes in the fruit maturing process. These four phases were green hard berry, green soft berry, pink soft berry and red soft berry. As shown in Figure 5B, different expression levels of the OPTs genes were found in the four different growth phases of the fruit, suggesting divergent functions of the OPT members in the maturing process.

Duplicated genes may have different evolutionary fates [54], which can be indicated by divergence in their expression patterns. Because tandem duplications may have governed the expansion of the OPT gene family, we also investigated the expression profiles of the duplicated OPT gene pairs identified above in Populus and Vitis. Our results show that none of the gene pairs share similar expression patterns (Figure 4 and 5), indicating that substantial neofunctionalization may have occurred during the subsequent evolution of the duplicated genes. It seems that the expression patterns of the paralogs have diverged during long-term evolution, suggesting functional diversification of the duplicated genes [55-58]. Such a process may increase the adaptability of duplicated genes to environmental changes, thus conferring a possible evolutionary advantage.

### Analysis of functional divergence

Next, we investigated whether amino acid substitutions in the highly conserved OPT domain could have caused adaptive functional diversification. Type-I functional divergence between gene clusters of the OPT family was estimated by posterior analysis using the program DIVERGE [59,60], which evaluate the shifted evolutionary rate and altered amino acid properties. Comparisons of thirty-five pairs of paralogous members and class OPT/class YSL proteins were carried out and the rate of amino acid evolution at each sequence position was estimated. Our results indicate that the coefficient of all functional divergence (θ) values between these groups or classes is less than 1 (Table 4). These observations indicate that there were significantly site-specific altered selective constraints on most members of the OPT family, leading to group-specific functional evolution after diversification. Moreover, critical amino acid residues responsible for the functional divergence were predicted based on site-specific profiles in combination with suitable cut-off values derived from the posterior probability of each comparison. The results indicate distinct differences in the number and distribution of predicted sites for functional divergence within each pair. For example, no critical amino acid site was predicted for the sequences in the Group 2/5, 4/5 and 9/11 pairs (Figure 1), while over 200 critical amino acids sites were predicted for Group 2/7, 2/8, 2/9, 2/10, 2/11, 4/7, 4/8, 4/9, 4/10, 4/11, 5/7, 5/8, 5/9, 5/10, 5/11, 6/7, 6/8, 6/9 and 6/10 pairs. Interestingly, when the OPT sequences in the OPT and YSL classes were compared, thirty-one critical amino acid sites were predicted for Group 6/11 pairs. When a cut-off value of 0.7 was applied, only four substitution sites were predicted, implying a lower evolutionary rate between the two pairs.

**Table 4:** Functional divergence estimated in OPT paralogs

Comparison	**θ**^1^	**SE**^2^	**LRT**^3^	**N(0.5)**^4^	**N(0.7)**^4^
Class YSL/Class OPT	0.8592	0.035797	576.1111	277	259
Group 2/Group 5	0.001	0.022361	0	0	0
Group 2/Group 6	0.4576	0.085419	28.69854	68	24
Group 2/Group 7	0.8136	0.111007	53.71832	280	272
Group 2/Group 8	0.9992	0.110362	81.9717	280	280
Group 2/Group 9	0.78	0.149011	27.40026	280	261
Group 2/Group 10	0.9992	0.085793	135.6435	280	280
Group 2/Group 11	0.8176	0.183891	19.76782	280	277
Group 4/Group 5	0.0682	0.096501	0.499466	0	0
Group 4/Group 6	0.2944	0.067278	19.14823	31	7
Group 4/Group 7	0.6616	0.07072	87.5186	262	187
Group 4/Group 8	0.8456	0.081838	106.7636	279	272
Group 4/Group 9	0.664	0.084659	61.51604	256	211
Group 4/Group 10	0.8112	0.063991	160.7009	278	255
Group 4/Group 11	0.6936	0.093695	54.80009	271	233
Group 5/Group 6	0.0162	0.106826	0.022997	3	2
Group 5/Group 7	0.6824	0.104652	42.5189	270	212
Group 5/Group 8	0.808	0.11001	53.9456	277	272
Group 5/Group 9	0.6632	0.138302	22.99485	272	220
Group 5/Group 10	0.8264	0.088168	87.85369	279	268
Group 5/Group 11	0.5936	0.149554	15.75407	263	239
Group 6/Group 7	0.7312	0.068942	112.4879	272	215
Group 6/Group 8	0.7312	0.076661	90.97606	271	222
Group 6/Group 9	0.58	0.075415	59.1481	233	157
Group 6/Group 10	0.7152	0.05871	148.3988	266	212
Group 6/Group 11	0.3656	0.089522	16.67828	31	4
Group 7/Group 8	0.1832	0.063668	8.279587	9	2
Group 7/Group 9	0.3552	0.07426	22.87913	30	11
Group 7/Group 10	0.2584	0.055745	21.4871	27	7
Group 7/Group 11	0.1264	0.071182	3.153215	2	1
Group 8/Group 9	0.1	0.06825	2.146829	1	0
Group 8/Group 10	0.168	0.060741	7.649795	4	2
Group 8/Group 11	0.2936	0.100349	8.560273	10	2
Group 9/Group 10	0.148	0.068012	4.73535	3	0
Group 9/Group 11	0.1058	0.1249	0.71754	0	0
Group 10/Group 11	0.156	0.088912	3.078442	2	0

^1^θ is the coefficient of functional divergence.

^2^SE: standard error.

^3^LRT is a likelihood ratio test.

^4^N(0.5) and N(0.7)means the numbers of divergent residues when the cut-off value is 0.5 and 0.7, respectively.

**Figure 6:**
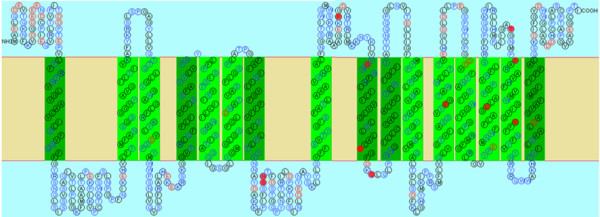
**Site specific profiles for evolutionary rate changes in Groups 7 and 9**. Eleven critical amino acid residues likely responsible for the functional divergence of these two groups were predicted and are shown in the filled red circles on the membrane topology model of VvOPT6, which was based on site-specific profiles combined with a suitable cut-off values (0.7) derived from the posterior probability of Group 7 and Group 9 comparison. Predicted membrane-spanning structure of VvOPT6 was generated by the computer topology prediction program SOSUI [77].

During a long period of evolution, the different evolutionary rates at specific amino acid sites within each pair might promote the functional divergence of OPT subfamilies. In Table 4, we also find that higher theta values (θ) exist in Group 2/8 (0.9992) and Group 2/10 (0.9992), indicating a higher evolutionary rate or site-specific selective relaxation between them. An example of the residues predicted to be functionally divergent was mapped onto the topology models of the Group 7/9 members (Figure 6). The predicted functional sites are not equally distributed throughout the OPT sequence, but are distributed in different α-helices and β-strands. The functions of these sites need to be experimentally verified. Thus, the results of the functional divergence analysis suggest that, because of the different evolutionary rates predicted at some amino acid sites, the OPT genes may be significantly divergent from each other in their functions. Perhaps, amino acid mutations have spurred the OPT family genes to evolve new functions after divergence and hence, functional divergence might reflect the existence of long-term selective pressures.

### Variable selective pressures among amino acid sites

**Figure 7:**
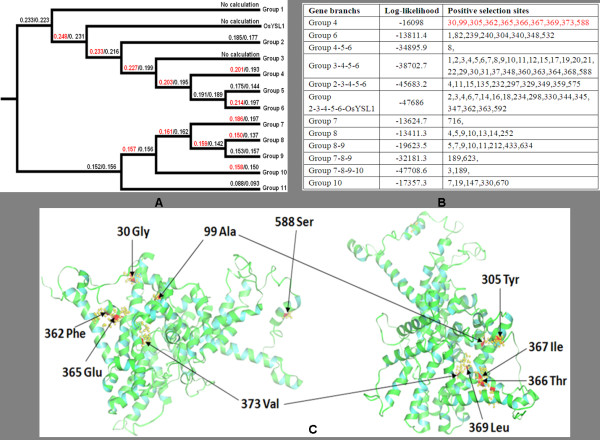
**Positive selection assessment of the OPT gene family in Arabidopsis, Populus, Vitis and rice**. **A**. Selection pressure (*K_a_/K_s_*) of the full-length OPT protein sequences for the different phylogenetic groups. Two different evolution models (M8/M7) were used. The M8 model was the only one that predicted the presence of positively selected sites (shown in red). **B**. Likelihood values and parameter estimates for the OPT genes predicted to undergo positive selection pressure as described in A. **C**. Detailed distribution of the positive selection sites of Group 4 predicted by the M8 model. Ten potential positive-selection sites are marked with arrows and shown in red in the tertiary structure of the PtYSL2 protein.

The *K_a_/K_s_*ratio measures selection pressure on amino acid substitutions. A *K_a_/K_s_*ratio greater than 1 suggests positive selection and a ratio less than 1 suggests purifying selection. The amino acids in a protein sequence are expected to be under different selective pressures and to have different underlying *K_a_/K_s_*ratios. To analyze positive or negative selection of specific amino acid sites within the full-length sequences of the OPT proteins in the different OPT groups, substitution rate ratios of nonsynonymous (*K_a_*) versus synonymous (*K_s_*) mutations were calculated with the Selecton Server http://selecton.tau.ac.il using a Bayesian inference approach [61]. The results show that the *K_a_/K_s_*ratios of the sequences from the different OPT groups are significantly different (Figure 7A). However, despite the differences in *K_a_/K_s_*values, all the estimated *K_a_/K_s_*values are substantially lower than 1, suggesting that the OPT sequences within each of the Groups are under strong purifying selection pressure and that positive selection may have acted only on a few sites during the evolutionary process. We performed the tests using the M8 (ω_s _> = 1), and M7 (beta) models. The selection model M7 does not indicate the presence of positively selected sites, whereas the M8 model does (Figure 7A and 7B). It is thus clear that, while most of the protein sequence is subjected to constant purifying selection, a few sites undergo positive selection. The detailed distribution of the positive-selection sites in Group 4 sequences as predicted by the M8 model are showed in Figure 7C. Further analyses indicate that six of the 10 positive selection sites in the Group 4 sequences are in α-helices (α1, α5 and α10). Interestingly, more than half of all the predicted positive-selection sites (Figure 7B) are in the β5 β-strand (2 sites) and in the α5 helix (4 sites). These observations suggest that positive selection pressure on the β-strands (β2, 4 and β5) and α-helices (α1, α5 and α10) might have accelerated functional divergence and the formation of the multiple subgroups. A few additional positively selected sites are distributed in other α-helices (α2-4 and α6-9), suggesting that these residues might be important in maintaining the conformational stability of the proteins.

## Conclusion

This study provides a comparative genome analysis addressing phylogeny, chromosomal location, gene structure, expression profiling, functional divergence and selective pressures of the OPT gene family in Populus and Vitis. Phylogenetic analyses revealed two well-supported classes in the OPT family, each of which can be further classified into 5 to 6 distinct groups. The exon/intron structure and motif compositions of the OPT genes and proteins are highly conserved in each class and in each of the groups, indicative of their functional conservation. The OPTs genes are non-randomly distributed across the Populus and Vitis chromosomes, and a high proportion of the OPT genes may be derived from tandem duplications. An additional comprehensive analysis of the expression profiles has provided insights into the possible functional divergence among members of the OPT gene family. Furthermore, functional divergence analyses suggest that significant site-specific selective constraints may have acted on most OPT paralogs after gene duplication, leading to subgroup-specific functional evolution. These data may provide valuable information for future functional investigations of this gene family.

## Methods

### Sequence retrieval and identification

To identify potential members of the OPT gene family in Populus and Vitis, we performed multiple database searches. Published Arabidopsis and rice OPT gene sequences [18,23] were retrieved and used as queries in BLAST searches against the Poplar Genome database http://genome.jgj-psf.org and the Genoscope Grape Genome database http://www.cns.fr. BLAST searches were also performed against the Poplar and Grape genomes at National Center for Biotechnology Information (NCBI, http://www.ncbi.nlm.nih.gov) and Phytozome http://www.phytozome.net.

WoLF PSORT http://wolfpsort.org[62] was used to predict protein subcellular localization. The TMHMM server http://www.cbs.dtu.dk/services/TMHMM/ was used to estimate the number of transmembrane helical domains. The isoelectric point (pI), molecular weight and grand average hydropathy (GRAVY) values were estimated using the ProtParam tool from ExPASy http://us.expasy.org/tools/protparam.html.

### Phylogenetic analyses of the OPT gene family

Multiple sequence alignments of the full-length protein sequences were performed using MUSCLE 3.52 [63], followed by manual comparisons and refinement. Gaps and ambiguously aligned regions were removed before phylogenetic analyses. ModelGenerator [64] was used to determine the substitution model and rate heterogeneity that best fit the OPT protein data. Phylogenetic analyses were performed with a maximum likelihood method using PhyML 3.0 [29] and a Bayesian inference method using PhyloBayes 3 [30]. The LG model of protein sequence substitution [65] and four gamma rate categories, as determined by ModelGenerator, were used for both maximum likelihood and Bayesian analyses. Bootstrap analyses for maximum likelihood analyses were performed using 100 pseudoreplicates. For Bayesian analyses, two independent runs were carried out with default settings until a maxdiff value = 0.27 was achieved to ensure chain equilibration (4,300 generations). The first 100 points were discarded as burn-in, and the posterior consensus was computed on the remaining trees. The topology depicted in Figure 1 was generated using PhyML.

### Inference of duplication time

Pairwise alignment of nucleotide sequences of the OPT paralogs was performed using MEGA 5 [66]. Alignments were performed using ClustalW (codons). The *K_a_*and *K_s_*values of the paralogous genes were estimated by the program K-Estimator 6.0 [67]. To better explain the patterns of macroevolution, estimates of the evolutionary rates were considered extremely useful. Assuming a molecular clock, the synonymous substitution rates (*K_s_*) of the paralogous genes would be expected to be similar over time. Thus, *K_s_*could be used as the proxy for time to estimate the dates of the segmental duplication events. The *K_s _*value was calculated for each of the gene pairs and then used to calculate the approximate date of the duplication event (T = *K_s_*/2λ), assuming clock-like rates (λ) of synonymous substitution of 1.5 × 10^-8 ^substitutions/synonymous site/year for Arabidopsis [48], 6.5 × 10^-9 ^for rice [68], 9.1 × 10^-9 ^for Populus [69], and 6.5 × 10^-9 ^for Vitis [70].

### Chromosomal location and gene structure of the OPT genes

The chromosomal locations of the OPT genes were determined using the Populus genome browser http://www.phytozome.net/poplar and Vitis genome browser http://www.genoscope.cns.fr/spip/Vitis-vinifera-e.html. Gene intron/extron structure information was collected from the genome annotations of Populus and Vitis from NCBI and Phytozome http://www.phytozome.net databases.

### Conserved motifs analyses

The program MEME http://meme.sdsc.edu[53] was used to identify motifs in the candidate Populus and Vitis OPT protein sequences. MEME was run locally with the following parameters: number of repetitions = any, maximum number of motifs = 30, and with optimum motif widths constrained to between 6 and 200 residues.

### Microarray analyses

The genome-wide microarray data of Populus published by Dharmawardhana and coworkers [71] were obtained from the NCBI Gene Expression Omnibus (GEO) with Accession Numbers GSE13043 and GSE21481. Probe sets corresponding to the putative Populus OPTs were identified on website http://genome.jgi-psf.org/. The microarray data for Vitis reported by Lund and coworkers [72] and Fennell [73] were obtained from GEO with Accession Numbers GSE11406 and GSE17502, respectively. The Plant Expression Database (PLEXdb, http://www.plexdb.org/index.php) [74] was also used for expression analyses. For genes with more than one set of probes, the median of expression values were used. Finally, the expression data were gene-wise normalized and hierarchically clustered based on Pearson coefficients with average linkage in the Genesis (version 1.7.6) program [75].

### Functional divergence analyses

To estimate the level of functional divergence and to predict amino acid residues responsible for functional differences in the OPT subfamilies, the coefficients of type-I functional divergence were calculated using the method suggested by Gu et al. [59,60]. The analyses were carried out with DINERGE (version 2.0). The method is based on maximum likelihood procedures to estimate significant changes in the site-specific shift of evolutionary rate or site-specific shift of amino acid properties after the emergence of two paralogous sequences. The advantage of this method is that it uses amino acid sequences and, therefore, is not sensitive to saturation of synonymous sites. Type-I functional divergence designates amino acid configurations that are highly conserved in gene 1 but highly variable in gene 2, or vice versa, implying that these residues have experienced altered functional constraints [59]. Coefficients of functional divergence that are significantly greater than 0 indicate site-specific altered selective constraints or radical shifts of amino acid physiochemical properties after gene duplication. Site-specific posterior analysis was used to predict amino acid residues that were crucial for functional divergence [45].

### Positive selection assessment

Identification of site-specific positive and purifying selection was calculated with the Selecton server http://selecton.tau.ac.il/, which uses a Bayesian inference approach for the evolutionary models [61,76]. *K_a_/K_s_*values are used to estimate the two types of substitutions events by calculating the synonymous rate (*K_s_*) and the non-synonymous rate (*K_a_*), at each codon site. The server implements several evolutionary models that describe in probabilistic terms how characters evolve. In this study, two of the evolutionary models (M8 and M7) were used. Each of the models uses different biological assumptions so that different hypotheses can be tested and the model that best fits the data can be selected. Briefly, M8 allows for positive selection operating on the protein. A proportion p_0 _of the sites are drawn from a beta distribution (defined in the interval 0 [1]), and a proportion p_1_(= 1-p_0_) of the sites are drawn from an additional category ω_s _(defined to be ≥ 1). Thus, sites drawn from the beta distribution are sites experiencing purifying selection, whereas sites drawn from the ω_s _category are sites experiencing either neutral or positive selection. The M7 model is similar to M8, except that it assumes only a beta distribution with no additional category. Thus, it allows mainly for purifying selection in the protein. These models all assume a statistical distribution to account for heterogeneous *K_a_/K_s_*values among sites. The distributions are approximated using eight discrete categories and the *K_a_/K_s_*values are computed by calculating the expectation of the posterior distribution [61].

## Authors' contributions

JC carried out the computational analyses and wrote the in-house program. JC and XH interpreted the results and wrote the manuscript. JC and YY were involved in planning of experiments. JH performed phylogenetic analyses and participated manuscript writing. XH revised the final version of the manuscript and headed the project. All authors read and approved the final manuscript.

## Supplementary Material

Additional file 1 **Figure S1**. Microarray based expression profiles of rice OPT genes across a variety of tissue or organs. Expression of OPT genes during developmental stages are presented as scatterplot at GENVESTIGATOR http://www.genevestigator.ethz.ch. The transcript levels are depicted by color scale representing log2 values. Red denotes high expression and green denotes low expression. OsYSL12 was not represented on the OS_51 K microarray. Click here for file

Additional file 2 **Table S1**. Pairwise identities between homologous pairs of OPT genes from Vitis, Populus, Arabidopsis and rice. Pairwise identities and sequence alignments of the 16 homologous pairs identified from the four species OPTs. Click here for file

Additional file 3 **Figure S2**. Chromosomal locations of the Arabidopsis OPT genes. The lines join the segmental duplicated homologous blocks. Click here for file

Additional file 4 **Figure S3**. Chromosomal locations of the rice OPT genes. The lines join the segmental duplicated homologous blocks that are indicated using the same colors. Click here for file

Additional file 5 **Figure S4**. Sequence logo and regular expression of the different motifs identified in the OPT gene family. Click here for file
