# Supplementary figures and images for: Analyses of the oligopeptide transporter gene family in poplar and grape
Source: BMC Genomics. 2011 Sep 26;12:465. doi: 10.1186/1471-2164-12-465 (PMC3188535; doi:10.1186/1471-2164-12-465)

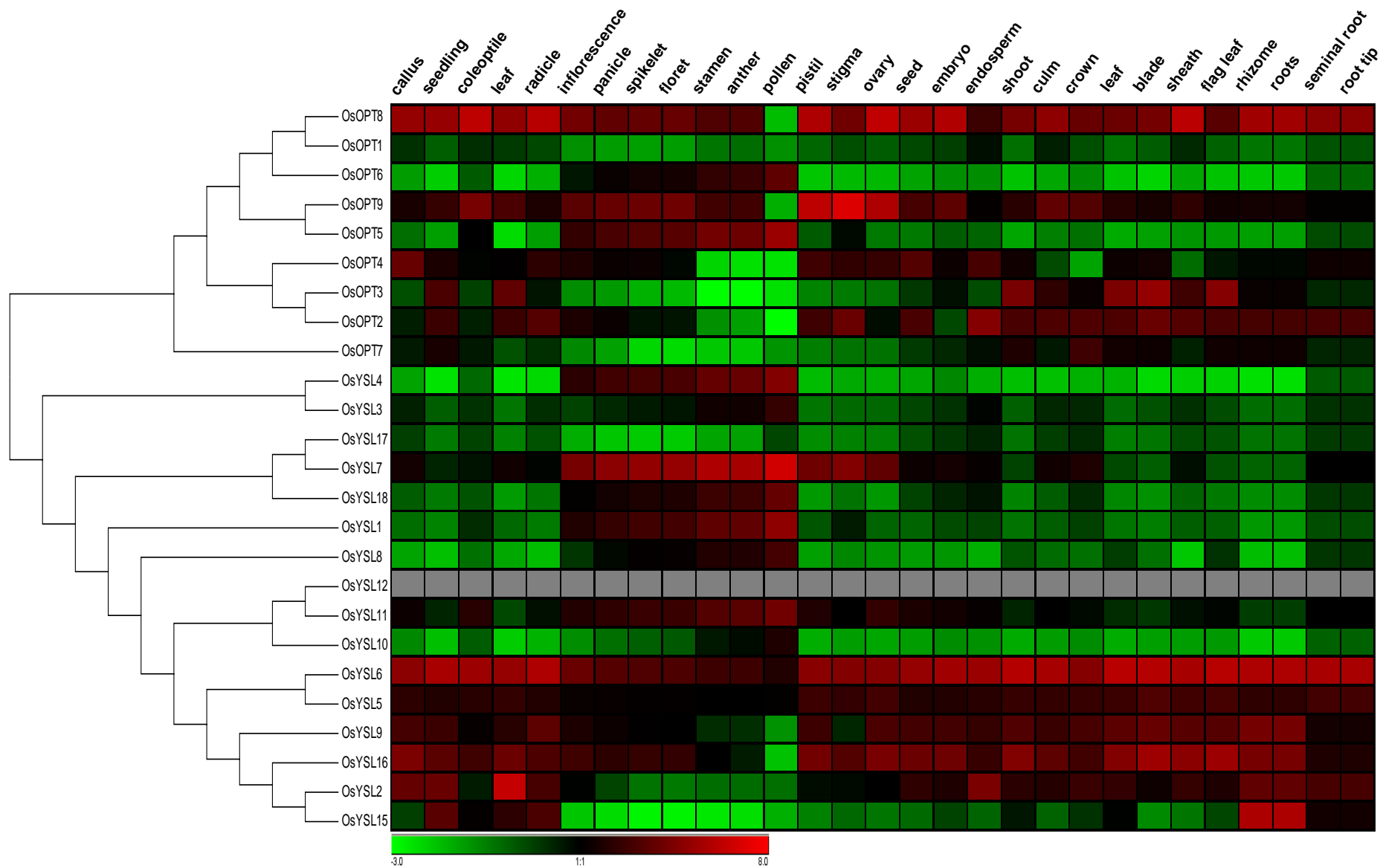

Supplement: Additional file 1 — Figure S1. Microarray based expression profiles of rice OPT genes across a variety of tissue or organs. Expression of OPT genes during developmental stages are presented as scatterplot at GENVESTIGATOR http://www.genevestigator.ethz.ch. The transcript levels are depicted by color scale representing log2 values. Red denotes high expression and green denotes low expression. OsYSL12 was not represented on the OS_51 K microarray. [file 1471-2164-12-465-S1.PDF]

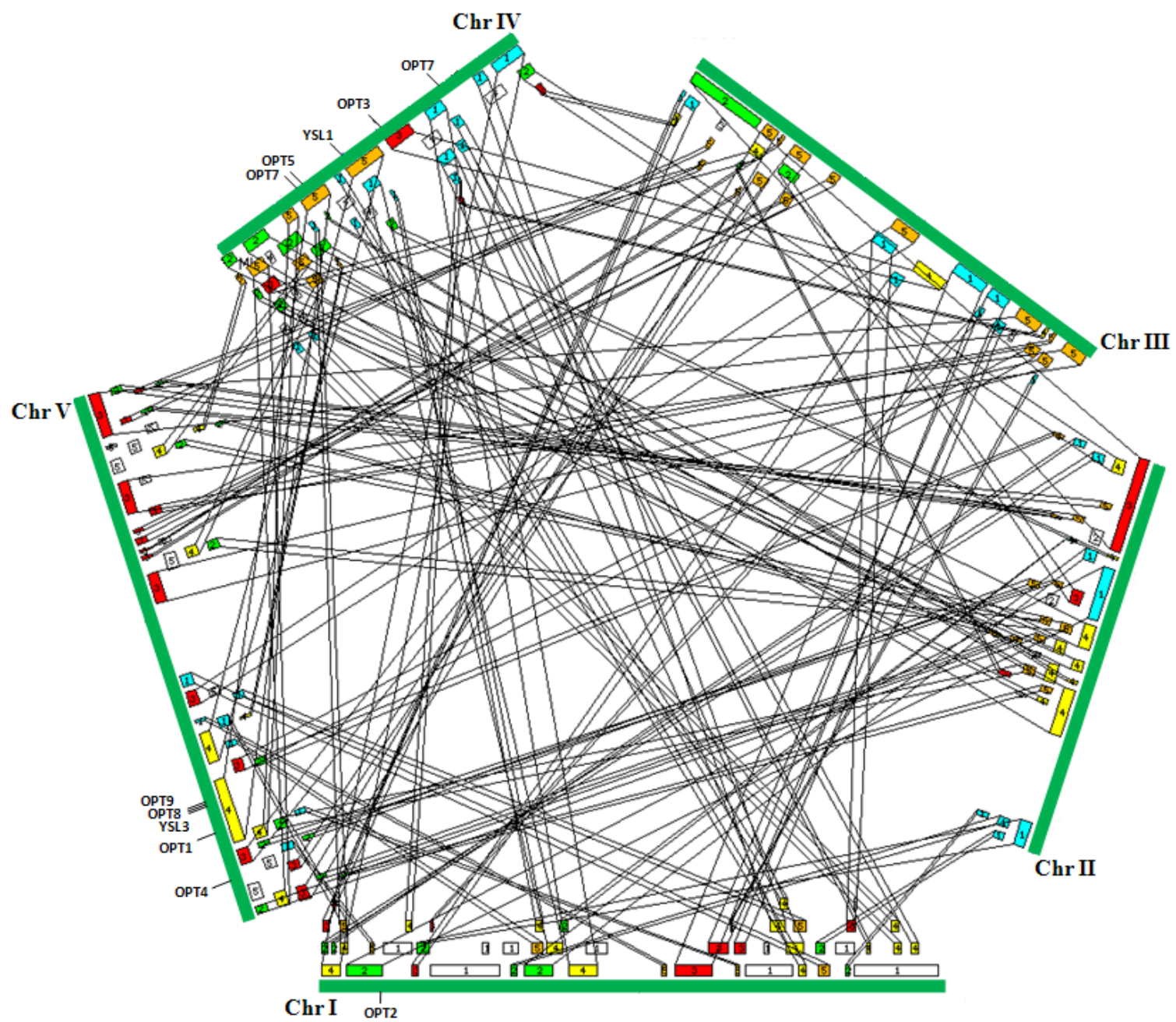

Supplement: Additional file 3 — Figure S2. Chromosomal locations of the Arabidopsis OPT genes. The lines join the segmental duplicated homologous blocks. [file 1471-2164-12-465-S3.PDF]

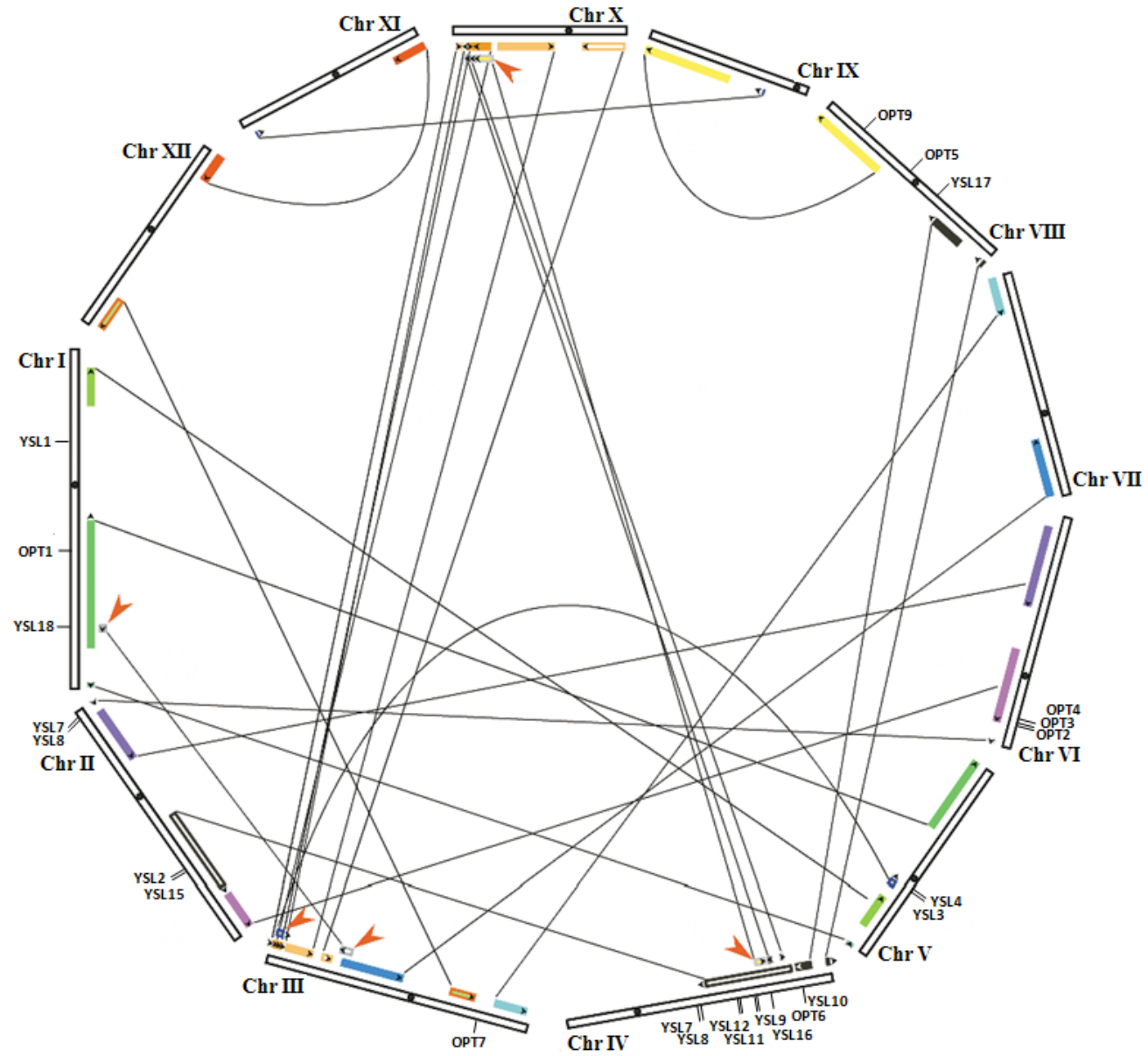

Supplement: Additional file 4 — Figure S3. Chromosomal locations of the rice OPT genes. The lines join the segmental duplicated homologous blocks that are indicated using the same colors. [file 1471-2164-12-465-S4.PDF]
