# Supplementary material for: Analyses of the oligopeptide transporter gene family in poplar and grape
Source: BMC Genomics. 2011 Sep 26;12:465. doi: 10.1186/1471-2164-12-465 (PMC3188535; doi:10.1186/1471-2164-12-465)
Supplement: Additional file 5 — Figure S4. Sequence logo and regular expression of the different motifs identified in the OPT gene family. [file 1471-2164-12-465-S5.DOC]

**Additional file 5**：**Sequence logo and regular expression of the different motifs identified in the OPT gene family.**


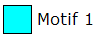


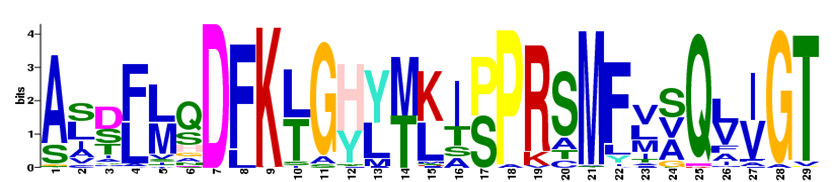


Regular expression: A[LS][DS][FL][LM][QS]DFK[LT]G[HY][YL][MT][KL][IT][PS]PRSMF[VL][SAV]Q[LV][IV]GT


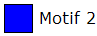


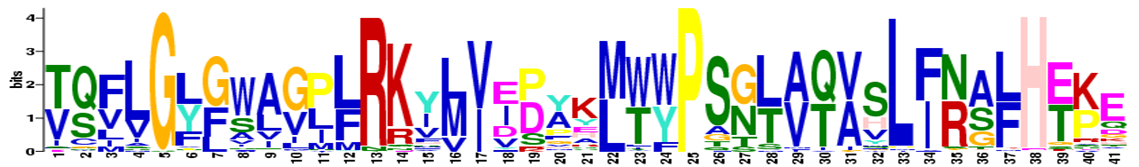


Regular expression:

[TV][QS][FV][LV]G[LY][GF][WS][AL][GV][PL][LF]RK[YI][LM][VI][EI][PD][AY]K[ML][WT][WY]PS[GN][LT][AV][QT][VA]SL[FI][NR][AS][LF]H[ET][KP]E


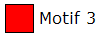


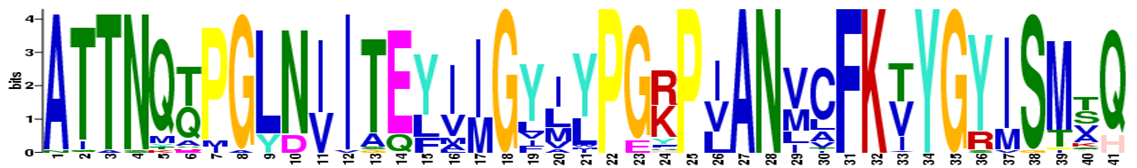


Regular expression:

ATTNQ[TQ]PGLN[IV]ITE[YL][IV][IM]GY[IL][YL]PG[RK]P[IVL]AN[VM]CFK[TV]YGYISM[TS]Q


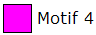


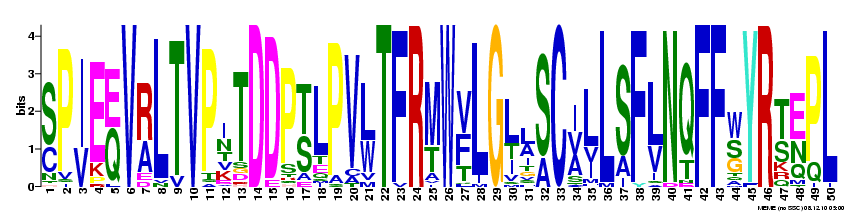


Regular expression:

[SC]P[IV]E[EQ]V[RA]LTVPITDDP[TS]LPV[LW]TFR[MT]W[VF]LGL[LA][SA]C[IVA]LLSF[LV]NQFF[WS]YR[TS][EN]PL


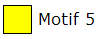


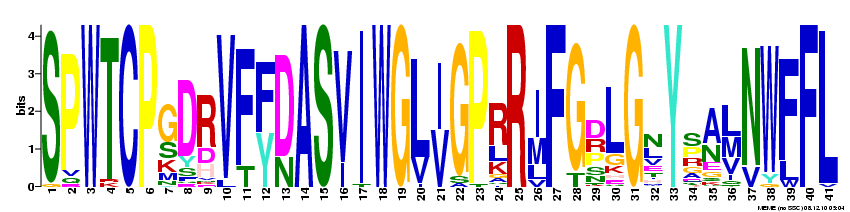


Regular expression:

SPWTCP[GS]DRVF[FY][DN]ASVIWG[LV][IV]GPRR[IM]FG[DPR]LG[NL]Y[SP][AN][LMV]NWFFL


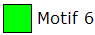


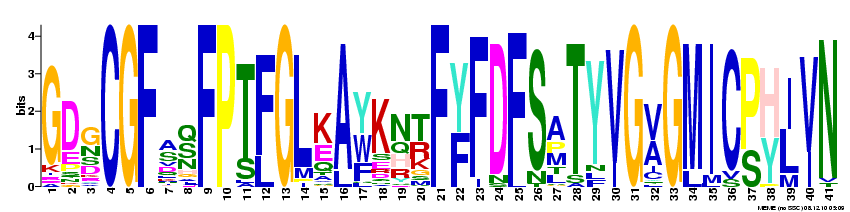


Regular expression:

GDGCGF[AS][QS]FPT[FL]GL[KE]A[YWF]KN[TR]F[YF]FDFSATYVG[VA]GMIC[PS][HY][IL][VI]N


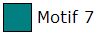


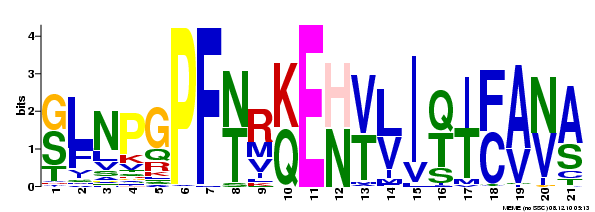


Regular expression:

[GS][LF]NPGPF[NT][RM][KQ]E[HN][VT][LV]I[QT][IT][FC][AV][NV][AS]


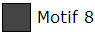


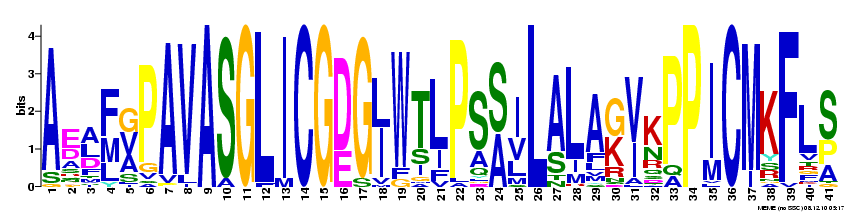


Regular expression:

A[ED][AL][FML][GVA]PAVASGLICG[DE]G[IL]WTLPS[SA][IV]L[AS]LA[GK][VI][KN]PPICMKFL[SP]


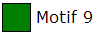


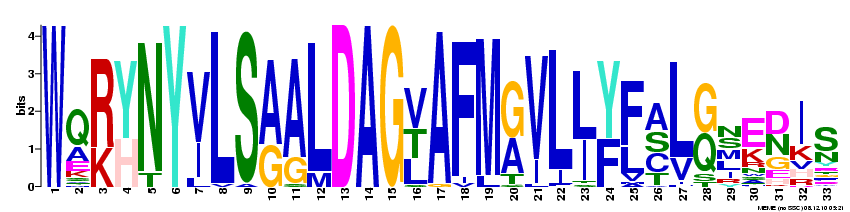


Regular expression:

WQ[RK][YH]NY[VI]LS[AG][AG]LDAG[VTL]AFM[GA]VL[LI][YF][FL][ASC]L[GQ]N[EK][DN]IS


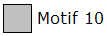


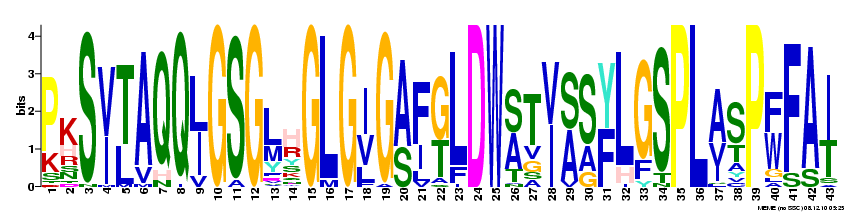


Regular expression:

PKS[VI][TL]AQQ[LI]GSG[LM]HGLG[IV]G[AS][FI][GT]LDW[SA]T[IV][SA][SA][YF]LGSPL[AV][ST]P[FW]FA[IT]


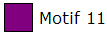


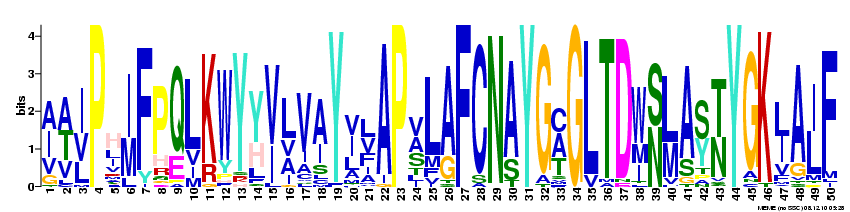


Regular expression:

[AIV][ATV][IVL]PH[IM]FP[QE]LKWY[YH][VI][LAV]V[AI]Y[IVL][LVF]AP[VAS]L[AG]FCNAYG[ACT]GLTD[WM][SN][LM]A[SY][TN]YGK[LI]A[IL]F


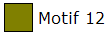


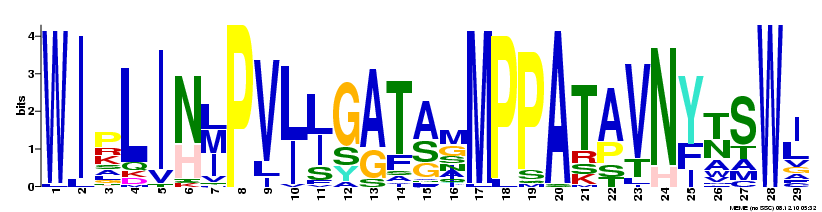


Regular expression:

WIPLI[NH][LIM]PV[LI][LI]G[AG]T[AS][MG]MPPAT[AP]VN[YF][TN]SW[IL]


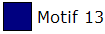


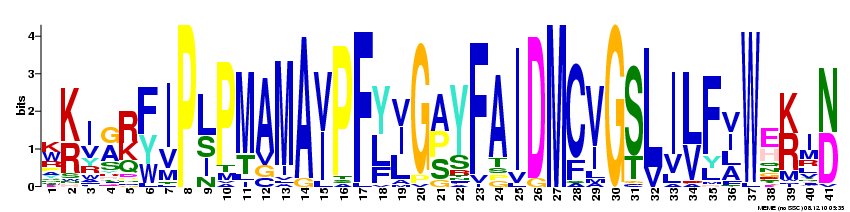


Regular expression:

[KW][KR][IV][AG][RK][FYW][IV]P[LSI]PMAMA[VI]PF[YL][IVL]G[APS]YFAIDMC[VI]G[ST]LI[LV]F[VIAL]W[EH][KR]I[ND]


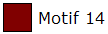


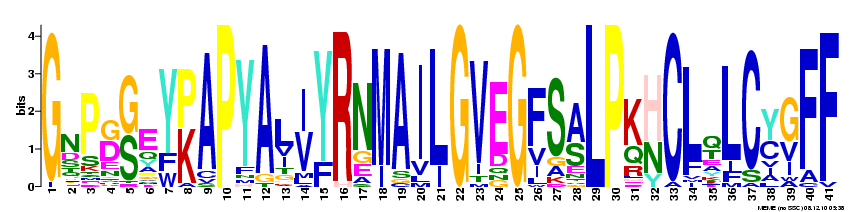


Regular expression:

GNP[DG][GS]EY[PK]APYAL[IV]YRNMAILGVEGFS[AS]LP[KQ][HN]CLQLC[YC][GV]FF


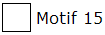


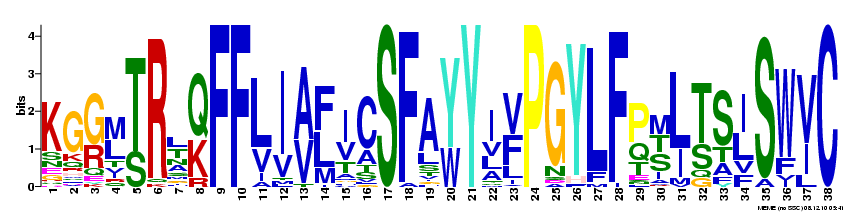


Regular expression:

KG[GR][ML][TS]RL[QK]FF[LV]I[AV][FL][IV]CSFA[YW]YI[VF]PGYLF[PQ][MST][LI][TS]S[IL]SW[VI]C


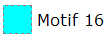


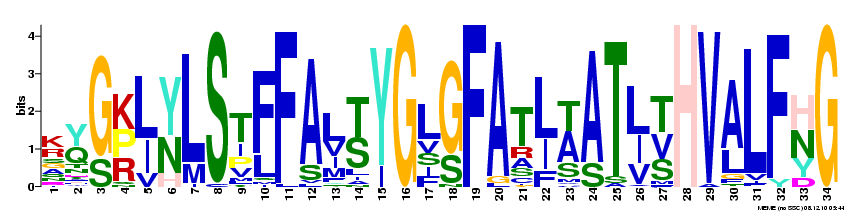


Regular expression:

[KR][YQ][GS][KPR][LI][YN]LST[FL]FA[LV][TS]YGL[GS]FAT[LI][AT]AT[LIV][TVS]HV[AL]LF[HNY]G


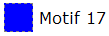


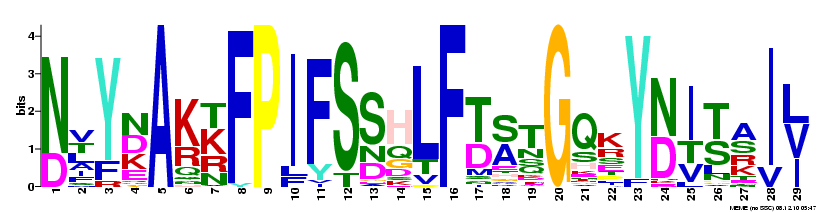


Regular expression:

[ND]VY[NDK]A[KR][KTR]FPIFSSHLF[TD][SA]TGQKY[ND][IT][TS][AS]I[LVI]


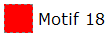


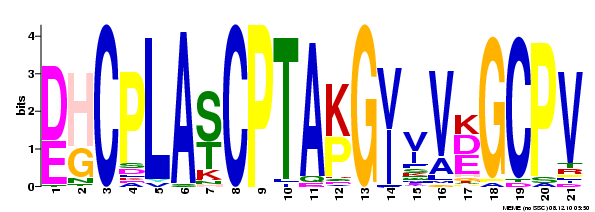


Regular expression:

[DE][HG]CPLA[ST]CPTA[KP]G[VI][VI]V[DKE]GCPV


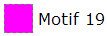


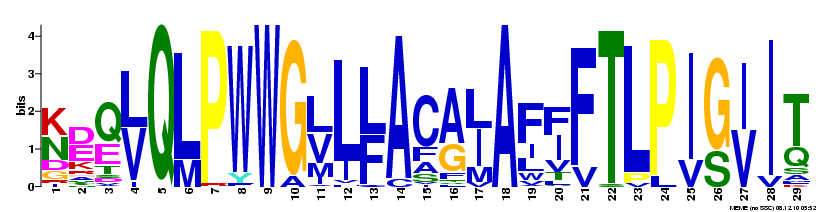


Regular expression:

[KN][DE][QE][LV]Q[LM]PWWG[LV]L[LF]AC[AG][LI]AF[FI]FTLP[IV][GS][IV]I[TQ]


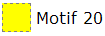


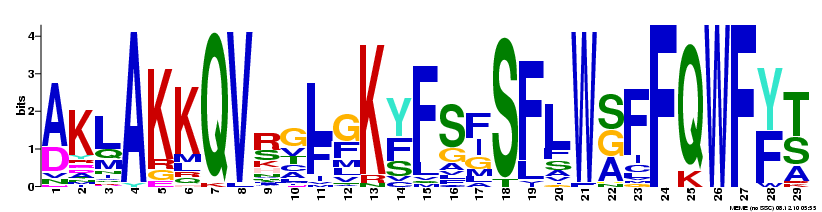


Regular expression:

[AD]KLAKKQV[RS]G[LF][GF]K[YF]FS[FI]SFLW[SGA]FFQWF[YF][TS]
